# Bronchoalveolar cytokine profile differentiates Pulmonary Langerhans cell histiocytosis patients from other smoking-related interstitial lung diseases

**DOI:** 10.1186/s12931-023-02622-z

**Published:** 2023-12-18

**Authors:** Silvia Barril, Paloma Acebo, Paloma Millan-Billi, Alfonso Luque, Oriol Sibila, Carlos Tarín, Abdellatif Tazi, Diego Castillo, Sonsoles Hortelano

**Affiliations:** 1 Respiratory Department, Institut de Recerca Biomèdica de Lleida (IRBLleida), Hospital Universitari Arnau de Vilanova-Santa María, Lleida, Spain; 2 Unidad de Terapias Farmacológicas, Instituto de Investigación de Enfermedades Raras (IIER), Instituto de Salud Carlos III (ISCIII), Madrid, Spain; 3 Respiratory Department, Hospital de la Santa Creu i Sant Pau, Sant Pau Biomedical Research Institute (IIB-Sant Pau, Barcelona, Spain; 4 Hospital Universitario Germans Trias I Pujol, Barcelona, Spain; 5 Unidad de Endotelio Funcional, Unidad Funcional de Investigación de Enfermedades Crónicas (UFIEC), Instituto de Salud Carlos III (ISCIII), Madrid, Spain; 6 R&D Department, Atrys Health, Barcelona, Spain; 7 R75006, INSERM U976 Human Immunology Pathophysiology and Immunotherapy (HIPI), Institut de Recherche Saint-Louis, Université Paris Cité, Paris, France; 8 National Reference Center for Histiocytoses, Department of Pulmonology, AP-HP, Hôpital Saint-Louis, Paris, France

**Keywords:** Pulmonary Langerhans cell histiocytosis, Interstitial lung disease, Bronchoalveolar lavage, Cytokines, Inflammatory profile

## Abstract

**Background:**

Pulmonary Langerhans cell histiocytosis (PLCH) is a rare interstitial lung disease (ILD) associated with smoking, whose definitive diagnosis requires the exclusion of other forms of ILD and a compatible surgical lung biopsy. Bronchoalveolar lavage (BAL) is commonly proposed for the diagnosis of ILD, including PLCH, but the diagnostic value of this technique is limited. Here, we have analyzed the levels of a panel of cytokines and chemokines in BAL from PLCH patients, in order to identify a distinct immune profile to discriminate PLCH from other smoking related-ILD (SR-ILD), and comparing the results with idiopathic pulmonary fibrosis (IPF) as another disease in which smoking is considered a risk factor.

**Methods:**

BAL samples were collected from thirty-six patients with different ILD, including seven patients with PLCH, sixteen with SR-ILD and thirteen with IPF. Inflammatory profiles were analyzed using the Human Cytokine Membrane Antibody Array. Principal component analysis (PCA) was performed to reduce dimensionality and protein–protein interaction (PPI) network analysis using STRING 11.5 database were conducted. Finally, Random forest (RF) method was used to build a prediction model.

**Results:**

We have found significant differences (p < 0.05) on thirty-two cytokines/chemokines when comparing BAL from PLCH patients with at least one of the other ILD. Four main groups of similarly regulated cytokines were established, identifying distinct sets of markers for each cluster. Exploratory analysis using PCA (principal component analysis) showed clustering and separation of patients, with the two first components capturing 69.69% of the total variance. Levels of TARC/CCL17, leptin, oncostatin M (OSM) and IP-10/CXCL10 were associated with lung function parameters, showing positive correlation with FVC. Finally, random forest (RF) algorithm demonstrates that PLCH patients can be differentiated from the other ILDs based solely on inflammatory profile (accuracy 96.25%).

**Conclusions:**

Our results show that patients with PLCH exhibit a distinct BAL immune profile to SR-ILD and IPF. PCA analysis and RF model identify a specific immune profile useful for discriminating PLCH.

**Supplementary Information:**

The online version contains supplementary material available at 10.1186/s12931-023-02622-z.

## Introduction

Pulmonary Langerhans cell histiocytosis (PLCH) is a rare interstitial lung disease (ILD) characterized by Langerhans-like cells accumulation resulting in granuloma formation and activation of inflammatory response [1, 2]. It affects young people with equal frequency in both genders and its development has been strongly related to smoking [1]. PLCH is also considered as a non-malignant neoplastic disorder associated with molecular abnormalities in the MAPK pathway [2–4]. Pathogenesis of PLCH remains unclear but numerous evidences indicate that PLCH lesions are characterized by the accumulation of Langerhans-like cells expressing CD1a and Langerin antigens at their surface admixed with inflammatory cells, including lymphocytes, eosinophils, macrophages and more rarely giant cells. PLCH granuloma is a dynamic process in which numerous cytokines, chemokines, growth factors and MMP have been found. Indeed, expression of tumor necrosis factor-α (TNF-α), Granulocyte–macrophage colony-stimulating factor (GM-CSF), transforming growth factor-β (TGF-β) and CCL20 has been described in PLCH lesions [1, 2].

Diagnosis of PLCH is often challenging, as definitive diagnosis requires the exclusion of other forms of ILD and surgical lung biopsy for histological examination and the identification of positive cells for CD1a or CD207 antigens [5]. Less invasive diagnostic tools will be helpful [6] but, up to now, a limited number of studies have explored potential biomarkers for diagnosis and prognosis of PLCH [7–9], and little data are available on their ability to differentiate PLCH from other ILDs. Here, we asked whether BAL fluid from PLCH patients exhibit a characteristic inflammatory profile that allow distinguish PLCH from other smoking related-ILD (SR-ILD), comparing the results with Idiopathic pulmonary fibrosis (IPF), as smoking is also considered to be a risk factor for the development of the disease. Additionally, the relationship between cytokine/chemokine profiles and lung function parameters was examined.

## Materials and methods

### Design

This study was an observational study conducted at the Respiratory Department of Hospital de la Santa Creu i Sant Pau, Barcelona from 2013 to 2019. The study was approved by Hospital de la Santa Creu i Sant Pau ethics committee (IIBSP-LAN-2013-39). All patients signed an informed consent before their inclusion in the study. The reporting of the results follows the STROBE guidelines [10].

### Population

Subjects were recruited from an ILD clinic. Only new referrals were considered. We invited to participate in the study those cases where a BAL was proposed as part of the diagnostic work-up. Patients were managed independently of the purpose of this study following institutional protocols based on national and international guidelines. Diagnosis was made after discussion of each case in the institutional ILD multidisciplinary meeting [11–15]. We included patients with PLCH, IPF and SR-ILD. Other inclusion criteria were: older than 18 years and signed informed consent. Exclusion criteria were: previous treatment for their lung condition including corticosteroids, immunosuppressive drugs or antifibrotics. Regarding diagnosis, PLCH cases were classified as definitive or probable depending on the availability of tissue to confirm the diagnosis. All patients with IPF had a definitive diagnosis based on current criteria (presence of UIP pattern on HRCT scan or histology in an adequate clinical context). Regarding SR-ILD, these groups compromised patients with desquamative interstitial pneumonia (DIP), respiratory bronchiolitis (BR), respiratory bronchiolitis with ILD (RB-ILD) and other fibrosing ILD patterns. Those with combined emphysema were classified as syndrome of combined pulmonary fibrosis and emphysema (CPFE).

### Data collection

ILD diagnosis, demographics, smoking history, High Resolution Computed tomography (HRCT) pattern and lung function tests (LFTs) were recorded and, if available, lung biopsy. Measurements of LFTs including forced vital capacity (FVC), forced expiratory volume in 1 s (FEV1) and diffusion capacity for carbon monoxide (DLCO) were acquired according to the Spanish Respiratory Society guidelines [6], using the predicted values for Mediterranean populations [16]. Airflow limitations were described as FEV1/FVC < 0.7. The lung function tests were done previous to the bronchoscopy, usually in the previous week. HRCT pattern was described following Fleischner recommendations [17].

BAL samples were collected from patients during routine diagnostic workup using 150-ml saline lavage with the bronchoscope wedged in the right middle lobe. Following current recommendations, to secure an optimal sampling, in all cases total volume retrieved was greater than 30% of the instilled volume, with a minimum of 10 mL. BAL samples were aliquoted after collection to avoid multiple freeze–thaw cycles. All samples were stored at − 80 °C until use.

### Inflammatory profile analyses

Inflammatory profiles were analyzed in BAL samples using the Human Cytokine Membrane Antibody Array ab133998 (Abcam, Cambridge, UK), which allows the detection of 80 human proteins (hereafter referred to as cytokines in order to simplify) encompassing cytokines/chemokines, growth factors, and matrix metalloproteinases (MMPs). Total protein concentrations of the samples were measured prior to sample incubation, showing similar ranges (coefficient of variation; CV ≤ 20%). Then, in accordance with manufacturer instructions, samples were diluted 1/5 (v/v) in 1X Blocking Buffer (provided in the Human Cytokine Membrane Antibody Array ab133998) to a final volume of 1 ml. Sample analysis was performed according to the manufacturer's instructions. Arrays images were processed with Chemi Doc XRS (Bio-Rad Laboratories, Hercules, CA). Densitometry measurements were performed with Quantity One software (Bio-Rad Laboratories). For every spot, the net intensity was determined by subtracting the average level of two negative controls. After background subtraction, negative signal intensities were assigned a value of 0. For multiple comparisons, average signal intensity of the six positive control spots was used to normalize the results from different membranes. Data are expressed as mean pixel density (MPD), or as fold change (FC) of each condition compared to PLCH samples. To identify target proteins displaying significant changes in expression, cut-off values of fold change (FC) of ≥ 1.5 or ≤ 0.50 for up- or downregulated proteins were used.

### Protein–protein interaction (PPI) network

Interactions between cytokines were analyzed using the Search Tool for the Retrieval of Interacting Genes/Proteins (STRING) software version 11.5 (http://string-db.org) [18]. STRING database provides PPIs from experimental interactions from different sources combining text and data mining approaches.

### ELISA assays

Levels of TARC and leptin was determined by ELISA in the BAL samples according to manufacturer instructions (Human CCL17/TARC DUOSET ELISA DY364 and Human LEPTIN QUANTIKINE ELISA KIT DLP00, R&D systems).

### Random forest (RF) classifier

Random forest (RF) classifier was used to assess the strength of association between the cytokine profile and the ILD group. Cytokine data (80 proteins) were filtered to those variables that were significantly expressed in one of the three groups (32 proteins). All data were processed using R studio (R 1.3.1075) and the **“**gdata”, “caret”, “purr”, “mlbench”, “randomForest” and “doParallel” packages. For RF algorithm, normalization of the dataset were not necessary. There were not variables with zero variance. Since redundancy of information decrease the prediction performances of models, strong correlations between variables (r > 0.9 and p < 0.05) were taken into account and 10 redundant variables were removed. Due to the imbalance of the groups (PLCH n = 7, SR-ILD = 16 and IPF = 13), we performed upsampling of the minority groups as the dataset is too valuable. Thus, some cases were replicated n-times in order to increase the number of the groups to the majority one. Additionally, in order to avoid an overfitting due to random multiplication of the profiles, we performed an independent tenfold upsampling creating different datasets and evaluating the RF algorithm with each of the datasets. Thus, the combined metrics of each fold constitute the final performance of the RF model. Data were divided into a training set with 75% of patients, and the remaining 25% were used for the test group in order to validate the predictor. First, we compared RF algorithm with the nearest neighbors method (k-Nearest Neighbors or KNN). RF model yielded the best accuracy and we decided to select it for further analysis. Finally, we tuned the number of predictors by a search grid, obtaining the best accuracy with combination of six features (Additional file 1: Fig. S1). Cross-validation with a k-fold = 50 was used in order to avoid bias during the training of the algorithm.

### Statistical analysis

All statistical analyses were performed with GraphPad prism software (version 9: GraphPad Software, San Diego, CA, USA) and R software (R Foundation for Statistical Computing, Vienna, Austria). All categorical variables are presented as numbers (percentages), parametric continuous variables are presented as mean ± SD, and nonparametric continuous variables are presented as median and interquartile ranges (IQR). When appropriate, variables were logarithmically transformed before statistical analysis to normalize the distribution and reduce the bias associated with extremely highly- and lowly- expressed chemokines and cytokines. Statistical comparisons of three or more groups were performed by using the two-way ANOVA with Tukey’s post-hoc or the Kruskal Wallis test for multiple comparisons. The Chi‐squared test or Fisher's exact test were used for categorical variables, as appropriate. Relationships between the Fold changes levels of the different cytokines as well as the lung function were examined using the Spearman’s rank correlation coefficient. Spearman’s rank correlation coefficients ≤ – 0.5 and ≥ 0.5 were considered as a strong correlation. A p-value of less than 0.05 was considered statistically significant. Unsupervised principal component analysis (PCA) was used to reduce dimensionality and identify trends in protein expression in ILD patients. Data was visualized by plotting the scores of the first two principal components (PC1 and PC2). Principal components were selected based on eigenvalues using mean-centring and normalization across samples. Loadings plot was used to identify the significant spectral regions responsible for sample clustering in the scores plot.

## Results

### Patient demographics and clinical characteristics

Thirty-six patients with interstitial lung diseases (ILD) were evaluated in the study including seven patients with pulmonary Langerhans cell histiocytosis (PLCH), sixteen with smoking related interstitial lung disease (SR-ILD) and thirteen with idiopathic pulmonary fibrosis (IPF). From those with PLCH, 2 had a definitive diagnosis based on lung biopsy samples. The remaining cases presented with a typical clinical-radiological picture compatible and were classified as probable PLCH following current guidelines. Regarding IPF, all patients included have a definitive diagnosis based in clinical and radiological findings (UIP pattern on HRCT scan). Concerning SR-ILD, two cases were diagnosed by lung biopsy. Cases were classified as follow: BR-ILD (n = 4), BR (n = 1), DIP (n = 4), CPFE (n = 7). Table 1 summarizes the demographic data, smoking habits, lung function and BAL differential cell counts of the ILD patients. As expected, PLCH patients were younger than the SR-ILD and IPF groups (median of 46 vs. 66 and 72 years old, P = 0.017 and P < 0.0001, respectively). Regarding CT features, the most prevalent finding was nodules that was presented in 42% cases and accompanied by cysts in 28.6%. Differences in gender between ILD groups were also observed, with a prevalence of males in SR-ILD and IPF groups (75% and 92%, respectively) and significant differences between PLCH and IPF patients (P = 0.0072). No significant differences in pulmonary function were found among the three groups, although the majority of patients with PLCH and SR-ILD exhibited airflow limitations (FEV1/FVC, % < 70% and DLCO, % < 75%). BAL fluid analysis showed an increase in polymorphonucleated leukocytes (PML) (> 5%) in all conditions, although no significant differences in the percentages of macrophages, neutrophils or lymphocytes among the different groups were found. Additionally, there was no correlation between the percentage of the different cell types and pulmonary function test (Additional file 1: Fig. S2).

**Table 1:** Demographic and clinical characteristics of ILD patients

	PLCH	SR-ILD	IPF
Number of patients	7	16	13
Predominant HRCT pattern (n, %)			
Nodules or micronodules	3 (42.8%)	–	–
Nodules and cysts	2 (28.6%)	–	–
Cysts	2 (28.6%)	–	–
Gender			
Male/female (n, %)	2/5 (29–71%)	12/4 (75–25%)	**12/1 (92–8%)**^**a**^
Age (yr)	46 (42–48)	**66 (52–72)**^**a**^	**72 (67–77)**^**a**^
Smoking status (n, %)			
Non-smokers	0 (0%)	0 (0%)	2 (15%)
Former smokers	1 (14%)	6 (40%)	2 (15%)
Current smokers	6 (86%)	10 (60%)	9 (69%)
Cigarettes (pack/yr)	25 (14–40)	47 (28–78)	35 (30–44)
Pulmonary function test			
FVC % predicted	89.71 (14.77)	93.81 (15.73)	**80.00 (9.6)**^**b**^
FEV1% predicted	71.71 (19.11)	82.56 (18.71)	82.85 (11.64)
FEV1/FVC, %	63.14 (12.23)	66.80 (11.28)	**75.86 (8.67)**^**b**^
DLCO, %	64.14 (15.32)	67.07 (19.21)	**51.23 (18.42)**^**b**^
BAL cell counts (%)			
Macrophages	70 (21.6)	71 (17.4)	59.75 (20.66)
PML	14.5 (12.67)	15.18 (10.09)	19.75 (18.86)
Lymphocytes	13 (9.84)	9.56 (7.49)	10.91 (5.4)

Values in bold indicate statistically significant results

Data are presented as %, median (interquartile range) or mean (SD) as appropriated

The p-values for differences between groups are indicated as a and b

FVC, forced vital capacity; FEV1, forced expiratory volume in 1 s; DLCO, diffuse capacity of the lung for carbon monoxide; BAL, bronchoalveolar lavage; PML, polymorphonuclear leukocytes; ILD, Interstitial lung disease; PLCH, Pulmonary Langerhans cell histiocytosis; SR-ILD, smoking related interstitial lung disease; IPF idiopathic pulmonary fibrosis

^a^P < 0.05 compared to PLCH

^b^P < 0.05 compared to SR-ILD

### Differential proteome inflammatory profile in ILD patients.

**Fig. 1:**
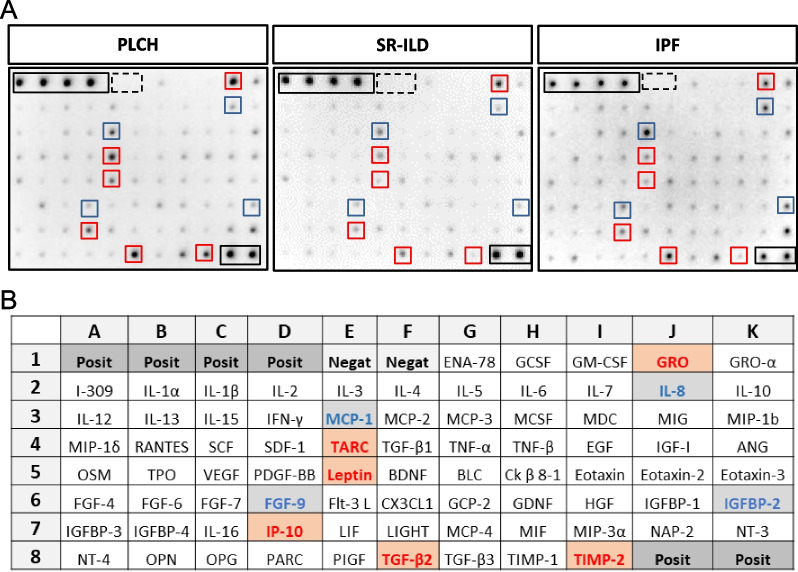
Differential cytokine/chemokine levels in BAL from ILD patients. **A** Human Cytokine Membrane Antibody Arrays were exposed to BAL from ILD patients and processed as per the manufacturer’s instructions. Images show representative membranes from each group of ILD patients. Black boxes denote positive internal controls and dashed black boxes denote negative internal controls. Representative cytokines with increased levels (red boxes) or decreased levels (blue boxes) in PLCH compared to SR-ILD or IPF are shown. **B** Schematic representation of the cytokine/chemokine spot positions on the membrane with respective internal controls. Rectangles filled with red background represents upregulated cytokines, and with blue background represents downregulated cytokines/chemokines in PLCH compared to SR-ILD or IPF

**Fig. 2:**
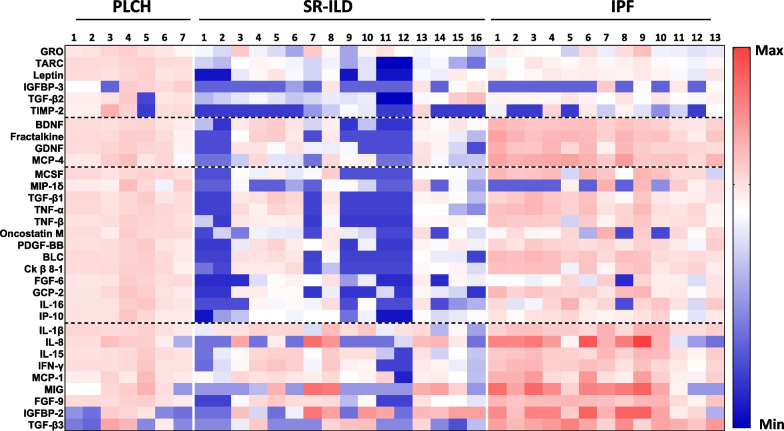
Protein inflammatory profile in BAL from ILD patients. **A** Heat map reconstruction of the selected chemokines, cytokines and growth factors differentially expressed (p < 0.05) in BAL from patients with ILD. Data are presented as Log_2_ fold changes (FCs) of protein levels. Higher and lower expression of heat maps is represented by red and blue, respectively. Each column represents data from independent samples. Each row represents a single protein. Dashed horizontal lines indicate groups of proteins similarly regulated in the different ILD pathologies

BAL of patients with different ILD (PLCH, SR-ILD and IPF) was analyzed using a Human Cytokine Membrane Antibody Array, in order to characterize the inflammatory profile. Representative images of the cytokines spot intensity signals, and MPD values are shown in Fig. 1 and Additional file 1: Table S1, respectively. BAL from PLCH patients exhibited significant differences in 32 cytokines (p < 0.05) when compared with at least one of the other ILD (Fig. 2 and Table 2). Four main groups of proteins could be established according to similar regulation in the different ILD pathologies (Table 2). Group I consisted in proteins significantly increased in PLCH compared to SR-ILD or IPF, including well-described mediators of inflammatory chemotaxis as growth-regulated oncogene (GRO)/CXCL1, thymus-and activation-regulated chemokine (TARC)/CCL17 or leptin; and metalloproteinases regulators as tissue inhibitor of metalloproteinases 2 (TIMP-2) (see Additional File 1: Fig. S3). Group II included proteins with significantly increased levels in PLCH vs SR-ILD, but whose values are significantly decreased compared to IPF patients, such as fractalkine/CX3CL1. Group III included proteins with significantly increased levels in PLCH vs SR-ILD, such as proinflammatory chemokines, as Oncostatin M (OSM), Fibroblast growth factor-(FBP)-6 and IFN-γ–induced protein (IP)-10/CXCL10. These proteins showed higher or lower levels in the PLCH patients compared to the IPF group but the changes observed were not statically different. Finally, group IV comprised several mediators of pulmonary fibrosis as IL-8/CXCL8, monocyte chemoattractant protein-1 (MCP-1)/CCL-2, monokine induced by IFN-γ (MIG)/CXCL9 and fibroblast growth factor-(FGF)-9, highly expressed in IPF patients but not in the other two groups (see Additional File 1: Fig. S3).

**Table 2:** Top proteins with significant differences in ILD pathologies

Protein	PLCH vs SR-ILD		PLCH vs IPF	
	FC	p value	FC	p value
Group I				
GRO (CXCL1)	1.822	0.010	1.934	0.006
TARC (CCL17)	3.861	< 0.0001	1.633	0.001
Leptin	3.103	< 0.0001	1.496	0.006
IGFBP-3	4.899	0.001	3.799	0.012
TGF-β2	2.266	< 0.0001	1.511	0.006
TIMP-2	7.029	0.004	3.072	0.028
Group II				
BDNF	1.897	0.017	0.598	0.002
Fractalkine (CX3CL1)	2.367	0.014	0.483	0.003
GDNF	2.586	0.007	0.586	0.028
MCP-4	2.676	0.033	0.373	0.001
Group III				
MCSF	2.955	0.003	0.643	ns
MIP-1δ	4.765	0.020	1.431	ns
TGF-β1	2.131	0.017	0.771	ns
TNF-α	2.633	0.005	0.644	ns
TNF-ß	2.628	0.019	0.831	ns
Oncostatin M (OSM)	3.623	0.009	1.120	ns
PDGF-BB	2.179	0.002	0.875	ns
BLC	2.977	0.044	0.611	ns
Ck β 8–1	2.794	0.023	0.696	ns
FGF-6	3.174	0.004	1.463	ns
GCP-2	3.720	0.016	0.701	ns
IL-16	3.462	0.003	0.954	ns
IP-10	3.561	0.000	1.205	ns
Group IV				
IL-1β	1.110	ns	0.574	0.009
IL-8 (CXCL8)	0.589	ns	0.133	< 0.0001
IL-15	1.455	ns	0.617	0.007
IFN-γ	1.466	ns	0.646	0.043
MCP-1 (CCL2)	1.393	ns	0.477	< 0.0001
MIG (CXCL9)	0.484	ns	0.158	0.010
FGF-9	1.714	ns	0.508	0.003
IGFBP-2	0.419	ns	0.159	0.009
TGF-β3	1.853	ns	0.265	0.005

Differential protein expression (Fold change) between groups (*p-*Value < 0.05) is shown

Statistical significance was determined by two-way analysis of variance (ANOVA)/Tukey’s multiple comparison test

FC, Fold Change; ILD, Interstitial lung disease; PLCH, Pulmonary Langerhans cell histiocytosis; SR-ILD, smoking related interstitial lung disease; IPF, idiopathic pulmonary fibrosis; ns, not significant

### Principal component analysis (PCA) identifies a different pattern of inflammatory proteins in ILD

**Fig. 3:**
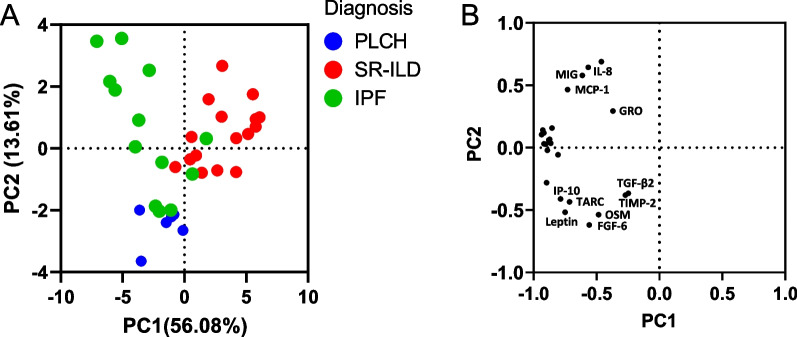
Principal Component Analysis of cytokine expression in ILDs. **A** PCA score plot shows separation of ILD based on first and second principal component scores. Each dot represents a sample projected in the two main principal components (PC1 and PC2); the dots are colored according to the group they belong to. **B** PCA loading plot shows the cytokines responsible for the differences between the groups. Labels are only shown for cytokines whose loading vectors on PC1 and PC2 exceeded a magnitude of 0.3

From the 32 cytokines that exhibited significant differences (p < 0.05) in BAL from PLCH patients (Fig. 2 and Table 2), we further selected the 24 cytokines that showed higher MPD values. PCA of the total patients (n = 36) and high expressed cytokines (n = 24 proteins) was performed to identify patterns in protein expression between the ILD. PCA revealed that the three groups were clustered separately with the greatest difference between PLCH and SR-ILD patients. The first two principal components on PCA score plot explained 69.69% of the variation, with a total variance of 56.08% for the first principal component and a 13.61% for the second principal component (Fig. 3A). Several cytokines mainly contributed to the separation of the groups as indicated by their position on the PC1 vs. PC2 loading plot (Fig. 3B). Interestingly, differences in levels of eleven cytokines including GRO/CXCL1, TARC/CCL17, leptin, TGF-β2, TIMP-2, OSM, FGF-6, IP-10/CXCL10, IL-8/CXCL8, MCP-1/CCL2 and MIG/CXCL9 were the main factors responsible for discriminating the groups. Of these, IL-8/CXCL8, MCP-1/CCL2 and MIG/CXCL9 were identified as significantly increased in IPF versus PLCH and SR-ILD, whereas TARC/CCL17, leptin, and IP-10/CXCL10 showed significant increases in PLCH group (Table 2).

### Protein–protein interaction and correlations of selected cytokines

**Fig. 4:**
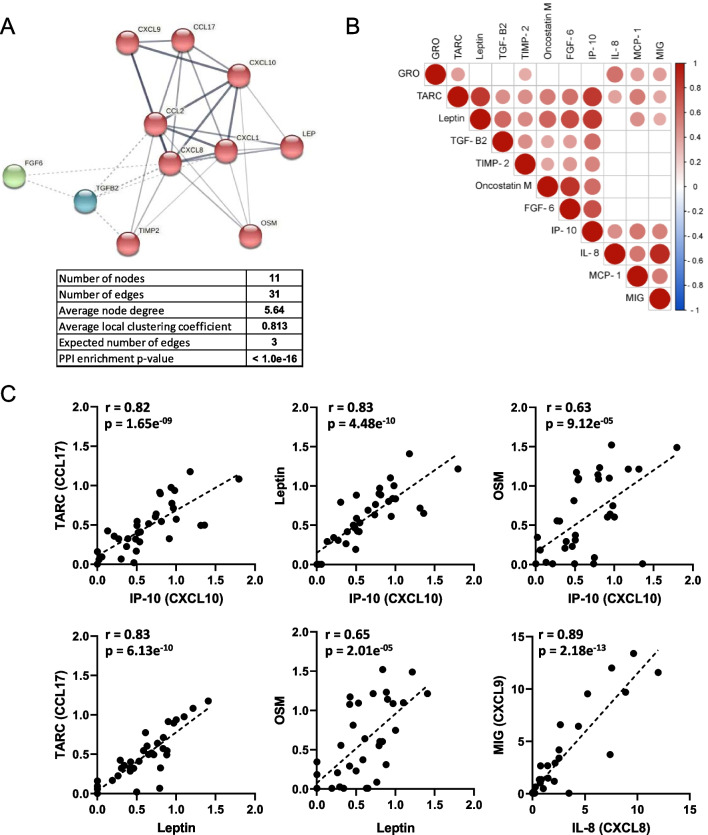
Protein–protein interaction and correlation analysis of the selected proteins. **A** Protein–protein interaction network visualized by STRING. In this network, nodes are proteins, edges indicate the number of interactions and the thickness of lines represents the strength of predicted functional interactions between proteins. The number of average interactions per node is indicated by the node degree. The clustering coefficient specifies the average node density of the map. Confidence parameter = 0.4. Three clusters (red, blue and green are shown (clusters *k*-means 3). **B** Correlation matrix depicts the Spearman’s correlation coefficient observed between differentially expressed proteins (Fold Change values). The intensity of the colors as well as the diameter of the circles give an indication of the degree of correlation between two cytokines and reflect the strength of spearman’s rho correlation coefficient, ranging from red (positive correlations) to blue (negative correlations). Only significant correlations are shown (p < 0.05). The white squares represent correlation coefficients that were not statistically significant. **C** Scatterplots depicting values of the different biomarker pairs

**Fig. 5:**
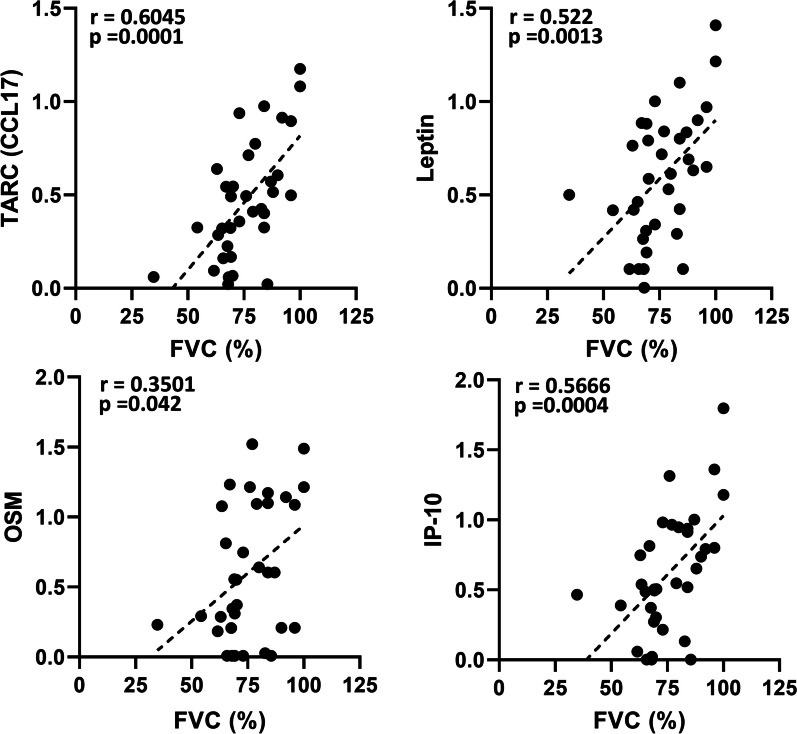
Correlation analysis of selected proteins with lung function parameters. Correlation coefficients were obtained by the Spearman rank method in PLCH, SR-ILD and IPF patients. Spearman’s correlation coefficient r and p values (two-tailed test) were shown in plots. FVC: forced vital capacity

To better understand the role of significant altered proteins in each disease, we conducted protein–protein interaction (PPI) network analysis using STRING 11.5 database. PPI analysis predicted close functional associations between the eleven selected proteins (Fig. 4A). All cytokines were found to be interconnected and three clusters were identified with one of them (red cluster), exhibiting strong association between of GRO/CXCL1, TARC/CCL17, IP-10/CXCL10, IL-8/CXCL8, MCP-1/CCL2 and MIG/CXCL9. A Spearman correlation analysis of fold changes was conducted to further explore the associations between cytokines (Fig. 4B). Most of the proteins exhibited positive correlations (Spearman r > 0.5, p = 0.05). Thus, the levels of IP-10/CXCL10 were highly correlated with the levels of TARC/CCL17, leptin and OSM; leptin was strongly correlated with TARC/CCL17 and OSM; and IL8/CXCL8 was strongly correlated with MIG/CXCL9 (Fig. 4C). Furthermore, when the analysis was conducted specifically in PLCH group, positive correlation among the most relevant cytokines (TARC, leptin, OSM and IP-10) was also observed (Additional File 1: Fig S4 and Table S2). Moreover, given that severe disease has been associated with altered cytokine expression, Spearman’s correlation analyses between cytokines and FVC values were also performed. Interestingly, TARC/CCL17, leptin, OSM and IP-10/CXCL10 levels were correlated with FVC (%) (Fig. 5). Additionally, we observed a negative correlation between cytokines and some of the lung parameters in PLCH patients, with significant results for TARC and leptin and the ratio FEV1/FVC (%) (r = − 0.909, p = 0.0120; r = − 0.8285, p = 0.0416, respectively) (Additional file 1: Fig S4 and Table S2).

**Fig. 6:**
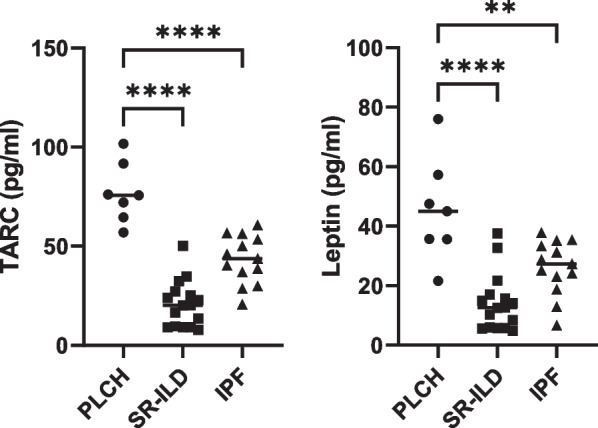
Measurements of the levels of TARC and leptin. Concentration of TARC and leptin was determined by ELISA in the BAL of patients with the different pathologies. Data are expressed as means ± SD. **p < 0.01, ****p < 0.0001

Finally, since TARC and leptin seem to play a relevant role in the inflammatory profile described in PLCH patients, we further evaluated the levels of these proteins by ELISA, in order to confirm the differences observed between groups. As observed in Fig. 6, PLCH patients exhibited significant higher levels of TARC and leptin.

### Development of a random forest predictor for discrimination of the groups

**Fig. 7:**
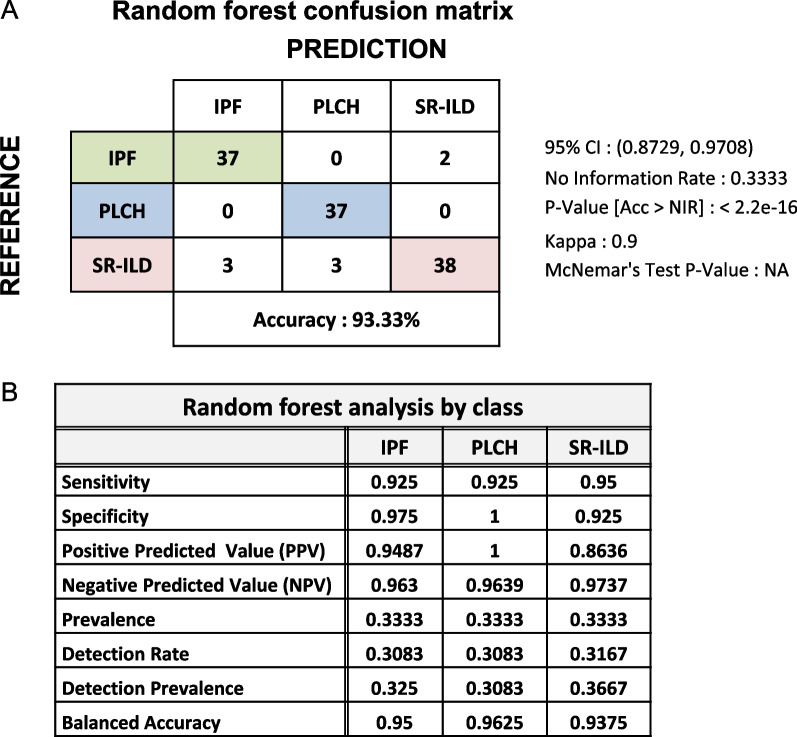
Classification model constructed using candidate cytokines. **A** Confusion matrix obtained by constructing RF classifier based on 6 candidates as features using a tenfold upsampling process. **B** Random forest analysis by class

Taken together, our data suggest that analysis of specific cytokine profiles in ILD patients may allow a more accurately classification. By using machine-learning algorithms (RF), we set out to build a classification model to discriminate the groups. From the 32 significantly expressed cytokines, 10 redundant variables were removed, taking into account strong correlations between variables (r > 0.9). As previously described (Additional file 1: Fig. S1), combination of six features was sufficient to obtain the best accuracy. The accuracy rates of the predictions are shown in Fig. 7. Confusion matrix showed a highly successful profile discrimination rate between the three groups which translates into an overall accuracy of 93.3% (Fig. 7A). Interestingly, the balance accuracy of the different classes was 0.9625, 0.9375 and 0.95 to PLCH, SR-ILD and IPF, respectively (Fig. 7B).

## Discussion

In the present study, we evaluated if the BAL fluid from PLCH patients exhibit a characteristic inflammatory profile. We quantified the alveolar levels of 80 analytes in patients with different ILDs including PLCH, SR-ILD and IPF. Our data provide strong evidence for an alveolar inflammatory profile that is differentially expressed in PLCH patients. We observed a significant change (p < 0.05) in 32 inflammatory proteins out of the 80 analyzed. Interestingly, the inflammatory pattern of BAL fluid from PLCH patients was characterized by up-regulation of 29 of these cytokines when compared with SR-ILD patients and 10 cytokines when compared to IPF. Despite its exploratory nature, our study identified key cytokines and inflammatory mediators that distinguish PLCH patients from SR-ILD and IPF patients. Moreover, we also found that some cytokines correlated with clinical outcome measures, suggesting their potential clinical importance in PLCH. Computational analysis (PCA and RF analysis) further show that PLCH patients could be differentiated based solely on inflammatory signature.

BAL is a diagnostic procedure recommended in patients with ILD. In PLCH, the goal is to exclude an infection and to look for the presence of CD1-positive cells (yielding more than 5%) which support the diagnosis of PLCH [19]. However, BAL analysis could offer more than cytological cell count. Cell gene expression, lung microbiota, microRNA signatures or proteomic analysis in BAL samples have shown promising results that could improve ILD characterization, including PLCH [20].

In our study, within the differential alveolar profile, we identified several inflammatory mediators with well-known roles in chemotaxis as GRO/CXCL1 or TARC/CCL17, regulators of matrix remodeling as TGF-β and metalloproteinases regulators as TIMP-2 and other mediators as leptin or IGFBP-3 that are not commonly associated with ILD.

Increased levels of GRO/CXCL1 are in line with the high number of neutrophils found in BAL, since this cytokine have strong neutrophil chemoattractant activity [21]. Furthermore, TARC/CCL17 has been recently described as a neutrophil-derived chemokine, mainly expressed and secreted in N2 neutrophils [22, 23]. Despite the fact that CXCL1 changes have been previously described in several lung diseases [24], less is known about the role of CCL17 in this context. CCL17 is a member of the CC chemokine family and has been traditionally associated with type 2 immune responses [24]. Cellular sources of this chemokine in the lung include not only bronchial and alveolar epithelial cells but also M2 macrophages and dendritic cells (DCs), including Langerhans cells [25]. Smoking related airway inflammation has been associated with elevated levels of CCL17 [26, 27] and increased concentrations of this chemokine have been found in BAL of IPF patients, suggesting its involvement in the pathophysiology of pulmonary fibrosis [28, 29]. Indeed, our data show that CCL17 levels increased in IPF patients compared to the SR-ILD group. Interestingly, PLCH patients showed significant higher levels of CCL17 compared to IPF and SR-ILD, suggesting a role for CCL17 in PLCH and probably reflecting a biased response toward an alternative activation of inflammatory cells. On the other hand, leptin has been described as a novel candidate to regulate pulmonary immune function [30]. In addition to its metabolic functions, leptin plays important roles in neutrophil and macrophage chemotaxis and association with smoking habits and lung dysfunction has been reported in several lung diseases [31, 32]. Here, we described for the first time that PLCH patients exhibited elevated levels of leptin. Furthermore, we have demonstrated positive correlations between leptin and TARC (r = 0.83, p = 6.13e−10) when compared across all groups. Interestingly, correlation of both cytokines was stronger when analysis was performed in the PLCH group (r = 0.926, p = 0.008). Our study also revealed that both cytokines were associated with lung dysfunction, since TARC/CCL17 and leptin levels were positively correlated with FVC values in all groups (r = 0.6045, p < 0.001 and r = 0.522, p < 0.001).

One further interesting finding was observed after analyzing the correlations between cytokines and lung function in PLCH group. A significant negative correlation between TARC and leptin with the ratio FEV1/FVC (%) was determined, suggesting the role of both cytokines in higher degrees of airways obstruction. Additionally, we have found important interactions with other cytokines as OSM or IP-10/CXCL10. In line with these findings, data in the literature has reported that OSM expression may be regulated by leptin [33]. Interestingly, OSM is elevated in various chronic lung diseases and has been involved in alternative programming of macrophages (M2 macrophages) [34], reinforcing the hypothesis of a biased inflammatory response.

In addition to inflammation, PLCH granuloma formation is accompanied by remodeling of the lung parenchyma. Elevated levels of TGF-β, MMPs, TIMPs (tissue inhibitors of MMPs), and IGFBP-3 have been shown to be at least partly responsible for the tissue destruction in several ILDs [35]. Here, we have shown that PLCH patients exhibited increased expression of TGF-β2, TIMP-2 and IGFBP-3 when compared to SR-ILD and IPF, probably as a reflection of tissue remodeling. Importantly, typical mediators of fibrosis as IL-8/CXCL8, MCP-1/CCL-2, and MIG/CXCL9 were highly expressed in IPF patients but not in the other two groups according to the different pathogenesis of these diseases.

Finally, confusion matrix showed a highly successful profile discrimination rate between the three groups which translates into an overall accuracy of 93.3%. This suggests that proteomic analysis of BAL samples could have a value as a diagnostic tool.

To our knowledge, this is the first study describing a specific inflammatory signature in PLCH patients. Despite the low sample size, we detected a consistent BAL inflammatory profile that could accurately discriminate PLCH from other ILDs as demonstrated by PCA analysis and machine learning studies. Nevertheless, due to the relatively small sample size, our results should be interpreted with caution and require further validation. The role of the identified cytokines in the pathogenesis of PLCH needs further research but we believe these results open a new window for improving the knowledge about this rare disease.

Additionally, we believe that further work could help to understand the role of the identified cytokines in the pathogenesis of PLCH.

## Conclusion

The findings of this study shows that patients with PLCH share a differential cytokine profile in BAL that could help to discriminate this entity from other ILDs. Further studies are needed to confirm the value of this signature in clinical practice and the link with the pathogenesis of the disease.

### Supplementary Information

**Additional file 1:** **Figure S1.** Variable selection for the RF classification model. **Figure S2.** Correlation matrix reporting Spearman’s correlation coefficient for BAL cells and lung function. **Table S1.** Cytokines and chemokines expression in BAL from ILD. **Figure S3.** Relative abundance of selected proteins for the established groups in ILD. **Figure S4.** Correlation matrix reporting Spearman’s correlation coefficient for cytokines and lung function in PLCH group. **Table S2.** Spearman´s correlation analysis for cytokines and lung function in PLCH group.

## Data Availability

All data reported this study are included in this published article and the Additional files.

## Abbreviations

BAL: Broncoalveolar lavage
CPFE: Combined pulmonary fibrosis and emphysema
DIP: Desquamative interstitial pneumonia
DLCO: Diffusion capacity for carbon monoxide
FGF: Fibroblast growth factor
FC: Fold change
FEV1: Forced expiratory volume in 1 s
FVC: Forced vital capacity
GM-CSF: Granulocyte–macrophage colony-stimulating factor
(GRO)/CXCL: Growth-regulated oncogene
HRCT: High Resolution Computed tomography
ILD: Interstitial lung disease
IPF: Idiopathic pulmonary fibrosis
(IP)-10/CXCL10: IFN-γ–induced protein.
IQR: Interquartile ranges
LFTs: Lung function tests
(MCP-1)/CCL-2: Monocyte chemoattractant protein-1,
(MIG)/CXCL9: Monokine induced by IFN-γ
MPD: Mean pixel density
MMPs: Matrix metalloproteinases
OSM: Oncostatin M
PCA: Principal component analysis
PLCH: Pulmonary Langerhans cell histiocytosis
PPI: Protein–protein interaction
PML: Polymorphonucleated leukocytes
RB-ILD: Respiratory bronchiolitis with ILD
RF: Random forest
SR-ILD: Smoking related-ILD
(TARC)/CCL17: Thymus-and activation-regulated chemokine
TIMP-2: Tissue inhibitor of metalloproteinases 2
TGF-β: Transforming growth factor-β
TNF-α: Tumor necrosis factor-α
