# Supplementary material for: Bronchoalveolar cytokine profile differentiates Pulmonary Langerhans cell histiocytosis patients from other smoking-related interstitial lung diseases
Source: Respir Res. 2023 Dec 18;24:320. doi: 10.1186/s12931-023-02622-z (PMC10729426; doi:10.1186/s12931-023-02622-z)
Supplement: Supplementary file 1 — Additional file 1: Figure S1. Variable selection for the RF classification model. Figure S2. Correlation matrix reporting Spearman’s correlation coefficient for BAL cells and lung function. Table S1. Cytokines and chemokines expression in BAL from ILD. Figure S3. Relative abundance of selected proteins for the established groups in ILD. Figure S4. Correlation matrix reporting Spearman’s correlation coefficient for cytokines and lung function in PLCH group. Table S2. Spearman´s correlation analysis for cytokines and lung function in PLCH group. [file 12931_2023_2622_MOESM1_ESM.docx]

**Additional File 1: Fig. S1. Variable selection for the RF classification model.** Search grid shows that the optimal selection with the corresponding highest accuracy (0.9905) of a predictive result after 10-fold cross-validation was obtained with six variables

**Additional File 1: Fig. S2.** **Correlation matrix reporting Spearman’s correlation coefficient for BAL cells and lung function.** The intensity of the colors as well as the diameter of the circles give an indication of the degree of correlation and reflect the strength of spearman’s rho correlation coefficient, ranging from red (positive correlations) to blue (negative correlations). Only significant correlations are shown (p < 0.05). The white squares represent correlation coefficients that were not statistically significant.

**
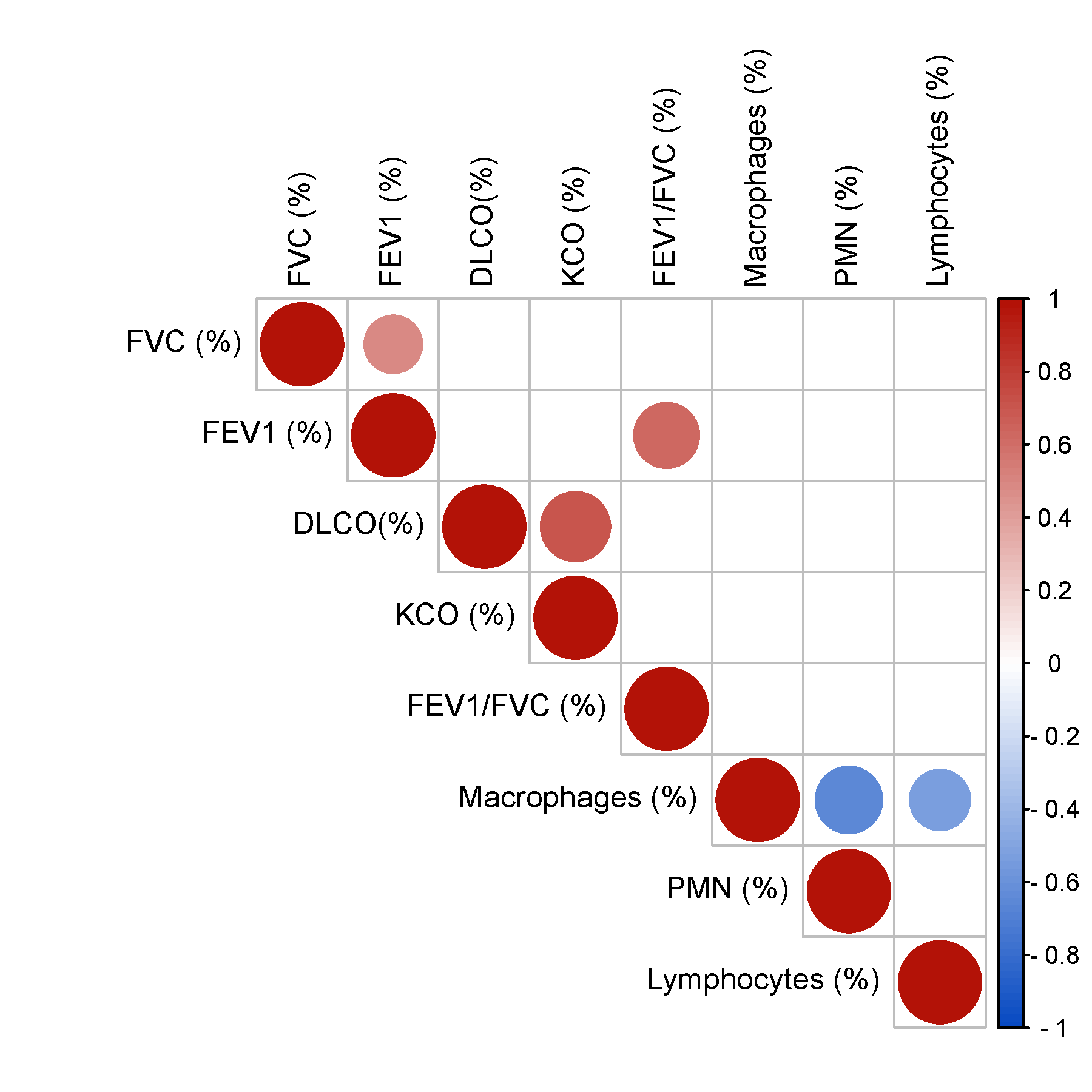
**

**Additional File 1: Table S1.** **Cytokines and chemokines expression in BAL from ILD.** Cytokine and chemokine levels are expressed as Mean Pixel Density (MPD) ± SD, normalized to the average of positive controls. ND means non-detectable. ILD = interstitial lung diseases; PLCH = Pulmonary Langerhans cell histiocytosis; SR-ILD = smoking related interstitial lung disease; IPF= Idiopathic pulmonary fibrosis.

|  | **PLCH (n = 7)** | | | **SR-ILD (n = 16)** | | | | **IPF (n = 13)** | | |
| --- | --- | --- | --- | --- | --- | --- | --- | --- | --- | --- |
| ENA-78 | 0,032 | ± | 0,018 | 0,016 | ± | 0,032 | 0,090 | | ± | 0,058 |
| GCSF |  | ND |  | 0,009 | ± | 0,022 | 0,023 | | ± | 0,028 |
| GM-CSF |  | ND |  | 0,016 | ± | 0,029 | 0,021 | | ± | 0,038 |
| GRO | 0,501 | ± | 0,140 | 0,275 | ± | 0,237 | 0,328 | | ± | 0,259 |
| GRO-α | 0,062 | ± | 0,066 | 0,085 | ± | 0,142 | 0,138 | | ± | 0,182 |
| I-309 | 0,011 | ± | 0,012 | 0,015 | ± | 0,027 | 0,031 | | ± | 0,046 |
| IL-1α | 0,044 | ± | 0,020 | 0,045 | ± | 0,044 | 0,102 | | ± | 0,076 |
| IL-1β | 0,097 | ± | 0,039 | 0,087 | ± | 0,058 | 0,169 | | ± | 0,061 |
| IL-2 | 0,077 | ± | 0,033 | 0,072 | ± | 0,062 | 0,122 | | ± | 0,041 |
| IL-3 | 0,119 | ± | 0,054 | 0,065 | ± | 0,059 | 0,122 | | ± | 0,054 |
| IL-4 | 0,062 | ± | 0,022 | 0,034 | ± | 0,043 | 0,062 | | ± | 0,040 |
| IL-5 | 0,040 | ± | 0,022 | 0,019 | ± | 0,038 | 0,085 | | ± | 0,059 |
| IL-6 | 0,020 | ± | 0,010 | 0,015 | ± | 0,031 | 0,064 | | ± | 0,052 |
| IL-7 | 0,004 | ± | 0,004 | 0,023 | ± | 0,047 | 0,041 | | ± | 0,048 |
| IL-8 | 0,044 | ± | 0,033 | 0,075 | ± | 0,139 | 0,331 | | ± | 0,423 |
| IL-10 | 0,001 | ± | 0,002 | 0,025 | ± | 0,054 | 0,004 | | ± | 0,012 |
| IL-12 p40/p70 | 0,028 | ± | 0,022 | 0,019 | ± | 0,031 | 0,062 | | ± | 0,058 |
| IL-13 | 0,036 | ± | 0,020 | 0,041 | ± | 0,047 | 0,086 | | ± | 0,069 |
| IL-15 | 0,120 | ± | 0,049 | 0,082 | ± | 0,065 | 0,194 | | ± | 0,066 |
| IFN-γ | 0,118 | ± | 0,057 | 0,081 | ± | 0,073 | 0,183 | | ± | 0,071 |
| MCP-1 | 0,256 | ± | 0,169 | 0,184 | ± | 0,099 | 0,536 | | ± | 0,400 |
| MCP-2 | 0,098 | ± | 0,047 | 0,040 | ± | 0,049 | 0,100 | | ± | 0,059 |
| MCP-3 | 0,074 | ± | 0,036 | 0,028 | ± | 0,037 | 0,092 | | ± | 0,044 |
| MCSF | 0,088 | ± | 0,038 | 0,030 | ± | 0,034 | 0,137 | | ± | 0,075 |
| MDC | 0,052 | ± | 0,022 | 0,027 | ± | 0,041 | 0,081 | | ± | 0,057 |
| MIG | 0,019 | ± | 0,018 | 0,040 | ± | 0,071 | 0,121 | | ± | 0,103 |
| MIP-1b | 0,063 | ± | 0,046 | 0,065 | ± | 0,097 | 0,084 | | ± | 0,069 |
| MIP-1δ | 0,067 | ± | 0,042 | 0,014 | ± | 0,022 | 0,047 | | ± | 0,063 |
| RANTES | 0,130 | ± | 0,049 | 0,076 | ± | 0,059 | 0,187 | | ± | 0,075 |
| SCF | 0,149 | ± | 0,065 | 0,062 | ± | 0,055 | 0,157 | | ± | 0,057 |
| SDF-1 | 0,140 | ± | 0,070 | 0,067 | ± | 0,068 | 0,186 | | ± | 0,062 |
| TARC | 0,487 | ± | 0,219 | 0,126 | ± | 0,101 | 0,298 | | ± | 0,079 |
| TGF-β1 | 0,122 | ± | 0,057 | 0,057 | ± | 0,064 | 0,159 | | ± | 0,069 |
| TNF-α | 0,123 | ± | 0,057 | 0,047 | ± | 0,058 | 0,192 | | ± | 0,074 |
| TNF-β | 0,152 | ± | 0,058 | 0,058 | ± | 0,052 | 0,183 | | ± | 0,075 |
| EGF | 0,122 | ± | 0,056 | 0,052 | ± | 0,058 | 0,154 | | ± | 0,074 |
| IGF-I | 0,091 | ± | 0,054 | 0,056 | ± | 0,061 | 0,104 | | ± | 0,044 |
| Angiogenin | 0,079 | ± | 0,053 | 0,055 | ± | 0,120 | 0,047 | | ± | 0,086 |
| Oncostatin M | 0,116 | ± | 0,047 | 0,032 | ± | 0,036 | 0,103 | | ± | 0,090 |
|  |  |  |  |  |  |  |  | |  |  |
| Cont. |  |  |  |  |  |  |  | |  |  |
|  | **PLCH (n = 7)** | | | **SR-ILD (n = 16)** | | | | **IPF (n = 13)** | | |
| Thrombopoietin | 0,073 | ± | 0,039 | 0,038 | ± | 0,047 | 0,090 | | ± | 0,065 |
| VEGF | 0,124 | ± | 0,039 | 0,068 | ± | 0,061 | 0,178 | | ± | 0,059 |
| PDGF-BB | 0,144 | ± | 0,058 | 0,066 | ± | 0,059 | 0,164 | | ± | 0,048 |
| Leptin | 0,291 | ± | 0,091 | 0,094 | ± | 0,083 | 0,195 | | ± | 0,050 |
| BDNF | 0,161 | ± | 0,041 | 0,085 | ± | 0,079 | 0,269 | | ± | 0,075 |
| BLC | 0,120 | ± | 0,056 | 0,040 | ± | 0,049 | 0,196 | | ± | 0,065 |
| Ck β 8-1 | 0,108 | ± | 0,050 | 0,039 | ± | 0,042 | 0,155 | | ± | 0,068 |
| Eoxatin | 0,059 | ± | 0,030 | 0,031 | ± | 0,031 | 0,152 | | ± | 0,069 |
| Eoxatin-2 | 0,068 | ± | 0,032 | 0,034 | ± | 0,049 | 0,102 | | ± | 0,060 |
| Eoxatin-3 | 0,005 | ± | 0,008 | 0,021 | ± | 0,044 | 0,008 | | ± | 0,015 |
| FGF-4 | 0,019 | ± | 0,019 | 0,012 | ± | 0,027 | 0,051 | | ± | 0,073 |
| FGF-6 | 0,184 | ± | 0,074 | 0,058 | ± | 0,061 | 0,126 | | ± | 0,082 |
| FGF-7 | 0,080 | ± | 0,039 | 0,036 | ± | 0,048 | 0,103 | | ± | 0,071 |
| FGF-9 | 0,128 | ± | 0,055 | 0,075 | ± | 0,066 | 0,251 | | ± | 0,074 |
| Flt-3 Ligand | 0,081 | ± | 0,046 | 0,033 | ± | 0,042 | 0,115 | | ± | 0,052 |
| Fractalkine | 0,096 | ± | 0,050 | 0,041 | ± | 0,049 | 0,199 | | ± | 0,075 |
| GCP-2 | 0,154 | ± | 0,086 | 0,041 | ± | 0,045 | 0,220 | | ± | 0,101 |
| GDNF | 0,097 | ± | 0,050 | 0,038 | ± | 0,038 | 0,166 | | ± | 0,063 |
| HGF | 0,053 | ± | 0,035 | 0,022 | ± | 0,027 | 0,093 | | ± | 0,056 |
| IGFBP-1 | 0,029 | ± | 0,028 | 0,023 | ± | 0,042 | 0,067 | | ± | 0,051 |
| IGFBP-2 | 0,045 | ± | 0,053 | 0,107 | ± | 0,125 | 0,281 | | ± | 0,272 |
| IGFBP-3 | 0,064 | ± | 0,036 | 0,013 | ± | 0,023 | 0,017 | | ± | 0,031 |
| IGFBP-4 | 0,068 | ± | 0,040 | 0,023 | ± | 0,037 | 0,059 | | ± | 0,057 |
| IL-16 | 0,098 | ± | 0,053 | 0,028 | ± | 0,035 | 0,102 | | ± | 0,072 |
| IP-10 | 0,289 | ± | 0,118 | 0,081 | ± | 0,062 | 0,240 | | ± | 0,088 |
| LIF | 0,178 | ± | 0,087 | 0,099 | ± | 0,131 | 0,179 | | ± | 0,055 |
| LIGHT | 0,055 | ± | 0,040 | 0,020 | ± | 0,028 | 0,126 | | ± | 0,053 |
| MCP-4 | 0,043 | ± | 0,030 | 0,016 | ± | 0,018 | 0,116 | | ± | 0,043 |
| MIF | 0,067 | ± | 0,039 | 0,033 | ± | 0,029 | 0,123 | | ± | 0,057 |
| MIP-3α | 0,026 | ± | 0,035 | 0,017 | ± | 0,026 | 0,059 | | ± | 0,051 |
| NAP-2 | 0,098 | ± | 0,061 | 0,036 | ± | 0,044 | 0,058 | | ± | 0,052 |
| NT-3 | 0,191 | ± | 0,055 | 0,117 | ± | 0,119 | 0,268 | | ± | 0,188 |
| NT-4 | 0,040 | ± | 0,040 | 0,009 | ± | 0,020 | 0,001 | | ± | 0,002 |
| Osteopontin | 0,047 | ± | 0,032 | 0,041 | ± | 0,058 | 0,289 | | ± | 0,632 |
| Osteoprotegerin | 0,033 | ± | 0,030 | 0,014 | ± | 0,030 | 0,049 | | ± | 0,067 |
| PARC | 0,030 | ± | 0,031 | 0,013 | ± | 0,024 | 0,049 | | ± | 0,079 |
| PIGF | 0,038 | ± | 0,036 | 0,011 | ± | 0,023 | 0,053 | | ± | 0,069 |
| TGF-β2 | 0,602 | ± | 0,366 | 0,266 | ± | 0,351 | 0,398 | | ± | 0,096 |
| TGF-β3 | 0,026 | ± | 0,040 | 0,014 | ± | 0,021 | 0,099 | | ± | 0,052 |
| TIMP-1 | 0,174 | ± | 0,118 | 0,093 | ± | 0,092 | 0,241 | | ± | 0,143 |
| TIMP-2 | 0,140 | ± | 0,134 | 0,020 | ± | 0,047 | 0,046 | | ± | 0,055 |

**Additional File 1: Fig. S3. Relative abundance of selected proteins for the established groups in ILD.** Scatter dot plots represent Mean Pixel Density (MPD) values for each individual patient of selected proteins on the different groups. Median values (horizontal bars) and IQR of the median are also shown. Statistical significance was determined by one-way Kruskal–Wallis tests (Dunn’s post hoc test) *p<0.05, **p<0.01, ***p<0.001, ****p<0.0001.


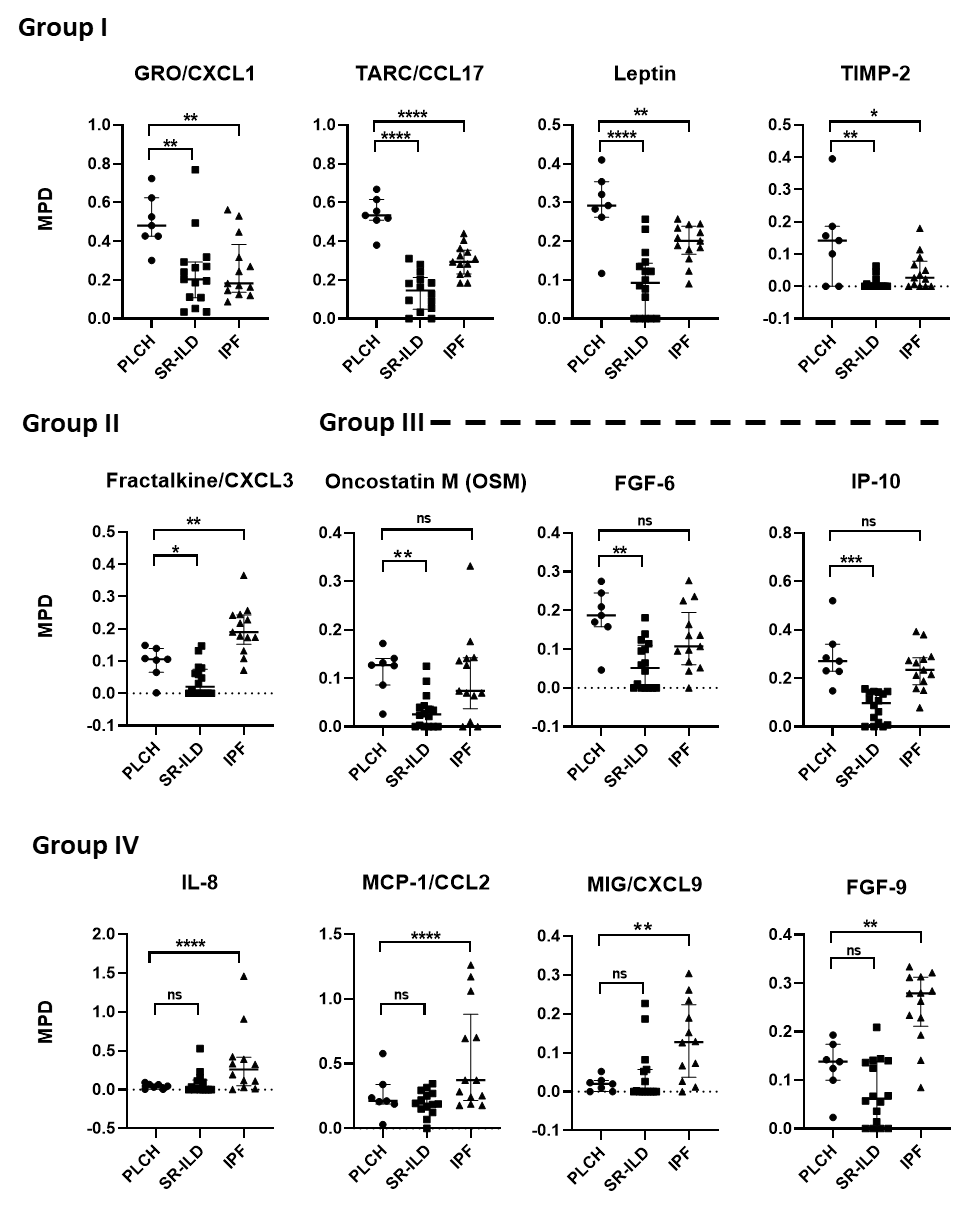


**Additional File 1: Fig S4. Correlation matrix reporting Spearman’s correlation coefficient for cytokines and lung function in PLCH group**. The intensity of the colors as well as the diameter of the circles give an indication of the degree of correlation and reflect the strength of spearman’s rho correlation coefficient, ranging from red (positive correlations) to blue (negative correlations). Only significant correlations are shown (p < 0.05). The white squares represent correlation coefficients that were not statistically significant.

**
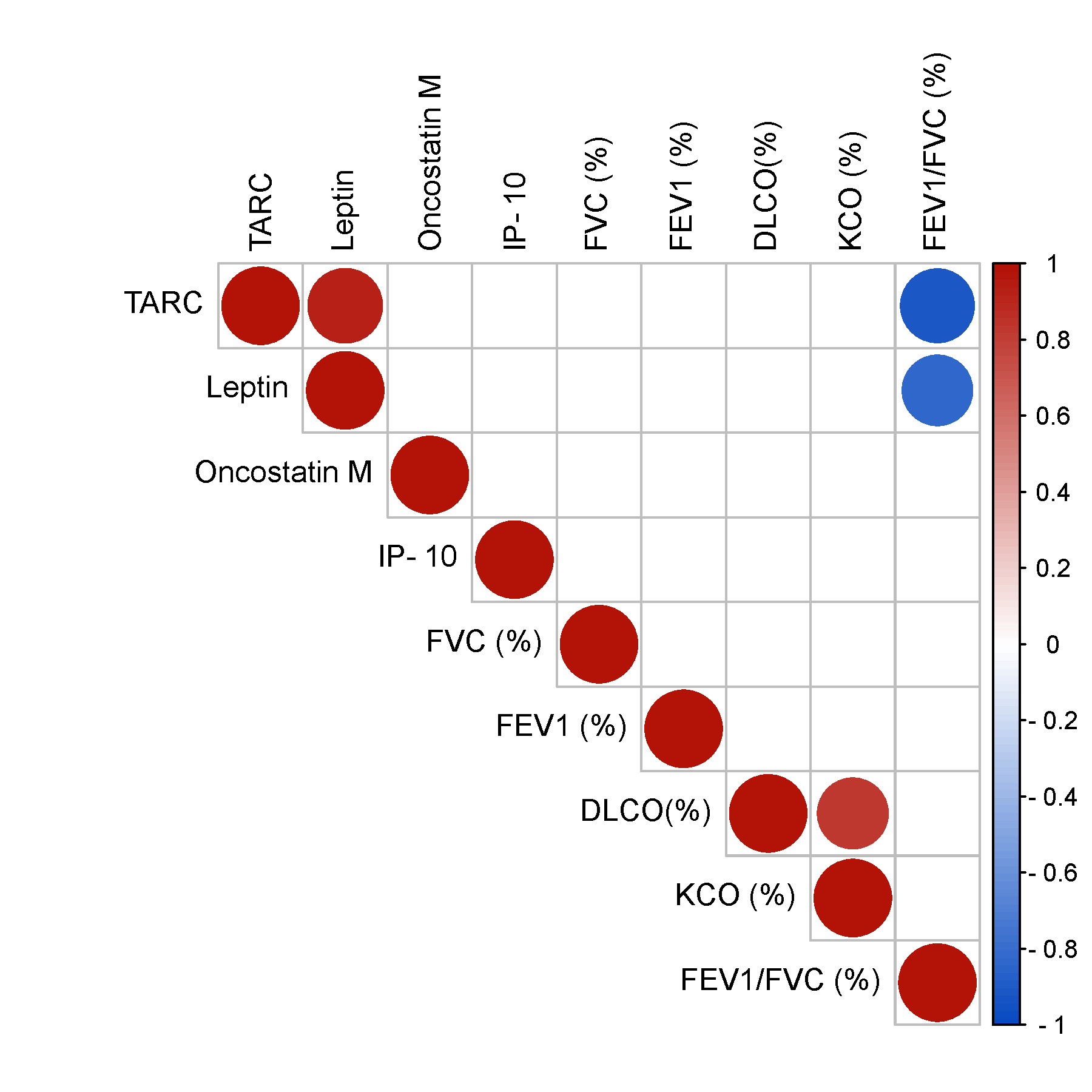
**

**Additional File 1: Table. S2. Spearman´s correlation analysis for cytokines and lung function in PLCH group.** Correlation coefficients (r) were obtained by the Spearman rank method in PLCH patients. P-value (two-tailed test) less than 0.05 means significant correlation. *p<0.05

|  | **TARC** | **Leptin** | **OSM** | **IP-10** | **FEV1/FVC (%)** |
| --- | --- | --- | --- | --- | --- |
| **TARC** | 1 | 0.93** | 0.65 | 0.83* | -0.76* |
| **Leptin** | - | 1 | 0.54 | 0.89* | -0.83* |
| **OSM** | - | - | 1 | 0.49 | -0.63 |
| **IP-10** | - | - | - | 1 | -0.52 |
| **FEV1/FVC (%)** |  |  |  |  | 1 |
